# Population-based study of chlamydial and gonococcal infections among women in Shenzhen, China: Implications for programme planning

**DOI:** 10.1371/journal.pone.0196516

**Published:** 2018-05-01

**Authors:** Zhen-Zhou Luo, Wu Li, Qiu-Hong Wu, Li Zhang, Li-Shan Tian, Lan-Lan Liu, Yi Ding, Jun Yuan, Zhong-Wei Chen, Li-Na Lan, Xiao-Bing Wu, Yu-Mao Cai, Fu-Chang Hong, Tie-Jian Feng, Min Zhang, Xiang-Sheng Chen

**Affiliations:** 1 Shenzhen Nanshan Center for Chronic Disease Control, Shenzhen, China; 2 Shenzhen Center for Chronic Disease Control, Shenzhen, China; 3 Chinese Academy of Medical Sciences & Peking Union Medical College Institute of Dermatology, Nanjing, China; 4 National Center for STD Control, Nanjing, China; University of Texas Health Science Center at San Antonio, UNITED STATES

## Abstract

This study was aimed to estimate the prevalences of chlamydia (CT) and gonococcal (NG) infections and explore risk factors associated with the CT infection among women in Shenzhen, China. We collected socio-demographic and clinical data from women (aged 20–60) and determined positivity of CT or NG by nucleic acid amplification test (NAAT) with self-collected urine specimens. We estimated prevalence of CT and NG and determined risk factors associated with CT infection. Among 9,207 participants, 4.12% (95% confidence interval [CI], 3.71%-4.53%) tested positive for CT and 0.17% (95% CIs, 0.09%-0.25%) for NG. Factors significantly associated with CT infection included being an ethnic minority (ethnicity other than Han China) (Adjusted odds ratio [AOR], 1.9; 95% CI, 1.2–3.0), using methods other than condom for contraception (AOR, 1.5; 95% CI, 1.2–1.8), having a history of adverse pregnancy outcomes (AOR, 1.4; 95% CI, 1.1–1.8), and experiencing reproductive tract symptoms in the past three months (AOR, 1.3; 95% CI, 1.0–1.7). we found that CT infection is prevalent among women in Shenzhen, China and associated with both demographic and behavioral factors. A comprehensive CT screening, surveillance and treatment programme targeting this population is warranted.

## Introduction

As major sexually transmitted infections (STIs), chlamydia caused by *Chlamydia trachomatis* (*C*. *trachomatis*, CT) and gonorrhoea caused by *Neisseria gonorrhoeae* (*N*. *gonorrhoeae*, NG) are important public health problems in many developed and developing countries [1–3]. According to the World Health Organization (WHO)’s most recent estimates, there were an estimated 131 million new cases or 127 million prevalent cases of CT and 78 million new cases of NG in 2012 [2]. If left untreated, chlamydia and gonorrhoea can cause significant morbidity, particularly in women, resulting in 30% risk to develop pelvic inflammatory disease (PID). PID can further cause serious reproductive and obstetric sequelae including ectopic pregnancy, tubal infertility, and chronic pelvic pain [4, 5]. As the most populous country in the world, China’s epidemic of STIs contributes significantly to the global burden of disease for CT and NG. In China, the National STI Surveillance Programme, as part of the “China Information System for Disease Control and Prevention” only tracks two notifiable STIs (syphilis and gonorrhoea) through an internet-based routine reporting network on a real-time basis [6]. Among the notifiable communicable diseases in China, STIs remain major causes of morbidity in which syphilis and gonorrhoea have ranked third and fifth respectively in term of the disease-specific reported incidence for more than a decade. Practically, the STI case reporting system monitors trends in the incidence of these diseases over time based on available patient data from health facilities. However, the asymptomatic nature of these infections, inadequate capacities of laboratories, and incomplete coverage of the surveillance programme could lead to a significant underreporting. In China, incidence of new STI infections is usually estimated from yearly case reports of patients attending clinics [7] while monitoring of prevalence among specific populations primarily relies on data from routine clinic-based screening programmes such as active screening for syphilis [8, 9] or ad hoc surveys among specific populations such as prevalence surveys of gonococcal and/or chlamydial infections among female sex workers [10]. An increasing number of studies on STI prevalence have been conducted among high-risk groups in China [10, 11] but the surveys of specific STIs among the general population, particularly chlamydia and/or gonorrhoea among women are limited [11, 12]. In this paper, we present a population-based study on prevalence and risk factors of CT and NG among women aged 20–60 years in Shenzhen City of China.

## Materials and methods

### Study area and population

This was a population-based survey conducted at the Nanshan District of Shenzhen City during March and August 2017. Nanshan District is located in the southwest of Shenzhen City and ranks the third in GDP (gross domestic product) among all of China’s district/county administrative level units. The current study was an additional survey to a local government public welfare programme that provides free screening for cervical cancer and breast cancer to all women. The local health authority of Nanshan District coordinated the programme via the administrative community networks in order to include a study sample representing all geographic regions and administrative levels. To encourage women in the community to participate in the survey, posters and leaflets were used to promote health education and community mobilization about CT and NG infection and the available screening services.

The study sample was purposely designed to be representative of the entire population in Nanshan District of Shenzhen. A probability proportional to size (PPS) sampling method was used for selection of sub-districts to include in the survey. Four (Nanshan, Nantou, Yuehai and Xili sub-districts, with a population of 0.22 million) of the total eight sub-districts in Nanshan District were finally selected as study sites to include in the survey. Women who were participating in the government-supported public welfare programme in these four sub-districts were then invited to participate in the study and assessed for eligibility. The study was based on recruitment of a convenience sample of women participating in a local government-supported public welfare programme using the eligibility criteria. Eligibility criteria included being a female resident aged 20–60 years and living locally in Nanshan District during the past 3 months.

### Questionnaire interview and specimen collection

After completing a written informed consent, eligible women were interviewed using a structured questionnaire to collect brief socio-demographic, and clinical information [11]. Interviews were conducted in a private room of the Nanshan Hospital of Maternal and Child Health Care. After completing the questionnaire, women were asked to provide a self-administered 3–5 mL first-catch urine specimen. A research nurse was assigned to check the integrity of questionnaire information and instruct participants in the performance of specimen collection. Urine specimens were collected using the Cobas^®^ urine specimen collection kit (Roche P/N 05170486190) according to the manufacturer’s instructions. The specimens were temporarily stored at 4°C in the local laboratory for a maximum 10 days before being transported to a central laboratory for testing.

### Laboratory assays

At the central laboratory, DNA was extracted and purified from the urine specimens by automated magnetism nucleic acid isolation method using the MagNA Pure 96 System (Roche, Switzerland) according to the manufacturer’s instructions. The extracted DNA was further evaluated for CT and NG based on polymerase chain reaction (PCR) of the Cobas 4800^®^ System (Roche, Switzerland) using Cobas^®^ 4800 CT/NG Amplification/Detection Kit. Diagnosis reagent and supplies were preserved under requested condition. Laboratory performance was run according to standard operating procedures (SOPs). CT or NG infection was defined as having a positive PCR for CT or NG accordingly.

### Statistical analyses

The study area is located along the coast where STI prevalence is typically higher than the national average [13]. In order to estimate an expected prevalence of 3.9% for CT and 0.12% for NG (a 50% higher than the prevalence reported in 2003 [11]) with α of 0.05 and a precision of 20% relatives to the expected prevalence, 2,367 and 7,908 women were needed respectively. Assuming a refusal and/or non-evaluable rate of approximately 12.5% (ranging 10%-15%), the estimated sample size was 2,705 for estimating prevalence of NG or 9,038 for prevalence of CT. Finally, the sample size was decided to be 9,100 women to be recruited and the sample size was further allocated proportionally into the four sub-districts using a ratio of 4.14% (9,100/220,000) to multiple the based on the baseline population size.

Questionnaire data and laboratory results were entered into Microsoft Office Excel (2013) database by one investigator and checked by another at the Nanshan Center for Chronic Disease Control. The Excel-format dataset was subsequently transferred to the IBM SPSS Statistics for Windows Version 20.0 (IBM Corp., Armonk, NY) for descriptive and inferential analyses. Categorical variables were compared using the chi-squared (χ^2^) test. Univariate analysis was used to determine association between variable and positivity for CT infection and odds ratio (OR) and 95% confidence interval (CI) were calculated. Variables with significance level of p ≤ 0.20 in univariate analyses were included in a multivariate logistic regression model to explore the association of variables with a specific outcome. Interactions between the independent variables were applied into the model analyses. Adjusted odds ratio (AOR) and its 95% CI were estimated. Values of p ≤ 0.05 were considered statistically significant. All relevant anonymous data are within the paper and its S1 Data.

### Ethics consideration

The Ethical Review Committee of the Nanshan Center for Chronic Disease Control reviewed and approved the study (Approval No. LL20170017). Patient’s questionnaires, and laboratory testing results were kept confidentially and data were entered into a computer anonymously. Participants who tested positive for CT and/or NG were contacted privately by the research team members for further treatment and other interventions for free at the STD clinic in the Nanshan Centre for Chronic Disease Control. Sex partner notification was conducted and free testing for sex partners was followed by free treatment of infected partners.

## Results

### Participant characteristics

Out of the 9,566 women who were interested in completing the survey, 9,249 (96.7%) met eligibility criteria and provided informed consent. A total of 9,207 (99.5%) participants who completed the questionnaire survey and provided urine specimens for successfully detecting CT and NG by PCR were finally included for data analyses. Socio-demographic characteristics of these participants are shown in Table 1. All participants were Chinese nationals and the majority (97.0%) of participants were of Han ethnicity. The mean age was 40.22 years (standard deviation [SD], 7.31 years).

**Table 1:** Prevalences of *C*. *trachomatis* and *N*. *gonorrhoeae* by socio-demographic characteristic.

Variable	Number (%)	Prevalence of *C*. *trachomatis (%)*		Prevalence of *N*. *gonorrhoeae (%)*	
		Rate	95% Cis*	Rate	95% Cis*
Age group (years)					
20–29	706 (7.67)	34 (4.82)	3.24–6.40	2 (0.28)	0–0.67
30–49	7548 (81.98)	309 (4.09)	3.64–4.54	14 (0.18)	0.08–0.28
50–60	953 (10.35)	36 (3.78)	2.57–4.99	0	0
Sub-district (SD)					
SD-1	2060 (22.37)	82 (3.98)	3.14–4.82	3 (0.15)	0–0.32
SD-2	2406 (26.13)	101 (4.20)	3.40–5.00	7 (0.29)	0.08–0.50
SD-3	2043 (22.19)	86 (4.21)	3.34–5.08	1 (0.04)	0–0.13
SD-4	2698 (29.30)	110 (4.08)	3.33–4.83	5 (0.18)	0.02–0.34
Permanent register					
Yes	3895 (42.30)	148 (3.80)	3.20–4.40	6 (0.15)	0.03–0.27
No	5312 (57.70)	231 (4.35)	3.80–4.90	10 (0.19)	0.07–0.31
Level of education					
Secondary school or below	3351 (36.40)	138 (4.12)	3.45–4.79	7 (0.21)	0.06–0.36
High school	3309 (35.94)	149 (4.50)	3.79–5.21	4 (0.12)	0–0.24
College or above	2547 (27.66)	92 (3.61)	2.89–4.33	5 (0.20)	0.03–0.37
Marital status^a^					
Married	9144 (99.32)	377 (4.09)	3.68–4.50	16 (100)	0.80–1.20
Single	44 (0.48)	1 (2.27)	*0–12*	0	0
Others	19 (0.20)	1 (2.27)	*0–26*	0	0
Employment status					
Un-employed	2927 (31.79)	102 (3.48)	2.82–4.14	1 (0.03)	0–0.09
Employed	6280 (68.21)	277 (4.41)	3.90–4.92	15 (0.24)	0.12–0.36
Monthly income (Chinese Yuan)					
Less than 4,000	5471 (59.42)	222 (4.06)	3.54–4.58	8 (0.15)	0.05–0.25
4,000–8,000	3072 (33.37)	134 (4.36)	3.64–5.08	7 (0.22)	0.05–0.39
More than 8,000	664 (7.21)	23 (3.46)	2.07–4.85	1 (0.15)	0–0.44
Ethnicity group^b^					
Han	8928 (97.00)	357 (4.00)	3.59–4.41	14 (0.16)	0.08–0.24
Others	279 (3.00)	22 (7.88)	4.72–11.04	2 (0.72)	0–1.71
Total	9207 (100)	379 (4.12)	3.71–4.53	16 (0.17)	0.09–0.25

^*a*^ Others include the separated, divorced or widowed status;

^*b*^ Others primarily include Dong, Miao or Yao ethnicity.

* CIs, confidence intervals.

### Prevalence of infection with CT and NG

Among the 9,207 participating women, 379 and 16 were detected to be positive for CT and NG, giving an overall prevalence of 4.12% (95% CI, 3.73%-4.54%) for CT and 0.17% (95% CI, 0.11%-0.28%) for NG, respectively. As shown in Table 1, compared with those in older age groups, young women aged 20–29 years had the highest prevalence of CT infection (4.82%, 95% CI: 3.24%-6.40%), but prevalence differences of either CT or NG infection between age groups were not statistically significant. The prevalence rates of CT infection in sub-districts 1, 2, 3 and 4 were 3.98% (95% CI, 3.14%-4.82%), 4.20% (95% CI, 3.40%-5.00%), 4.21% (95% CI, 3.34%-5.08%) and 4.08% (95% CI, 3.33%-4.83%), respectively, and not statistically significantly different each other.

### Factor associated with infection of CT

In the univariate analyses, six variables were associated with chlamydial infection at P < 0.20 (Table 2). In multivariate analyses using these six variables as independent variables and potential interactions between these variables, the following factors were found to be significantly associated with CT infection after adjusting for potential confounding factors: being an ethnicity other than Han majority (AOR = 1.9, 95% CIs = 1.2–3.0, P = 0.005), using methods other than a condom for contraception (AOR = 1.5, 95% CIs = 1.2–1.8, P = 0.001), having a RT symptom in the recent 3 months (AOR = 1.3, 95% CIs = 1.0–1.7, P = 0.02) and experiencing an adverse pregnancy outcome (AOR = 1.4, 95% CIs = 1.1–1.8, P = 0.007), Table 2.

**Table 2:** Factors associated with positivity of *C*. *trachomatis*: univariate and multivariate logistic regression analyses.

Factor	Univariate analysis		Multivariate analysis	
	OR* (95% CI)**	P value	AOR*** (95% CI)	P value
Employment status^a^				
Un-employed	Reference		Reference	
Employed	1.3 (1.0–1.6)	0.04	1.3 (1.0–1.6)	0.06
Ethnicity group^b^				
Han	Reference		Reference	
Others	2.1 (1.3–3.2)	0.02	1.9 (1.2–3.0)	0.005
Number of pregnancies^c^				
None	Reference		Reference	
One time	0.6 (0.3–1.2)	0.12	0.7 (0.4–1.4)	0.32
Two or more times	0.8 (0.4–1.6)	0.55	1.0 (0.5–1.8)	0.79
Adverse pregnancy outcome^d^				
No	Reference		Reference	
Yes	1.5 (1.2–2.0)	<0.001	1.4 (1.1–1.8)	0.007
Contraception method^e^				
Condom	Reference		Reference	
Others	1.4 (1.1–1.7)	0.003	1.5 (1.2–1.8)	0.001
RT symptoms in recent 3 months^f^				
No	Reference		Reference	
Yes	1.4 (1.1–1.8)	0.003	1.3 (1.0–1.7)	0.02

^*a*^ Employment status refers to whether the participant has a job at the moment of the survey;

^*b*^ Others primarily include Dong, Miao or Yao ethnicity;

^*c*^ Pregnancy refers to any gestation for at least 1.5 months;

^*d*^ Adverse pregnancy outcomes include having any lifelong experience of abortion, premature delivery, ectopic pregnancy or infertility;

^*e*^ Other contraception methods refer to those other than condom, including oral pills, intrauterine devices (IUD), injectables, and vaginal ring;

^*f*^ RT symptoms refer to self-notified reproductive tract symptoms including vaginal symptoms including abnormal vaginal discharge, and leucorrhoea increase in quantity.

* OR, odds ratio;

** CIs, confidence intervals;

*** AOR, adjusted odd ratio.

## Discussion

To our knowledge, this is the largest population-based sample to date to estimate prevalence of CT and NG infections in China. The sample size (n = 9,207) and participation rate (99.5%) are much higher than those in the first nationwide population-based survey in 1999 and 2000, in which 1,738 women were included with a participating rate of 69% [11] or the recently published survey in Hong Kong in which 535 women were included with a participating rate of less than 25% [12]. Together with use of rational assumptions for expected prevalence and its relative precision, large sample size and its allocation into sub-district community using the PPS sampling method as well as high participating rate ensures sufficient power for this study to accurately estimate the prevalences of CT and NG in women population in the study area.

Our findings indicate high prevalence rates of CT (4.12%) and NG (0.17%), as compared to the national rates of 2.6% and 0.08% (1999–2000) [11] or 1.4% and 0% in a recent city-wide population-based study in Hong Kong [12]. This CT prevalence is also higher than that reported in many high-income countries [14], such as 2.0% (95% CIs, 1.5%-2.5%) in the US [15], 1.5% (95% CIs, 1.1%-2.0%) in the UK [16], and 1.6% (95% CIs, 1.0%-2.5%) in France [17]. In our study, younger women had the higher prevalence of CT infection, which is consistent with previous literatures [11, 18]. However, the rate of 6.59% in our age-group of 20–24 years is consistent with the 6.5% reported in women aged 18–29 years from a national population-based study in Peru [19] but this rate is generally higher than that reported in the similar age-group of women in high-income countries including the UK (2.7%) [16], the US (4.7%) [20], the Netherlands (4.3%) [21], Croatia (5.3%) [22] and Norway (5.1%) [23]. Interestingly, our prevalence of NG infection is lower than that reported in high- or middle-income countries [17, 19] although the rate is generally low. Further studies on the reasons for this phenomenon of high CT but low NG prevalence in the general population are needed.

We evaluated socio-demographic and clinical characteristics associated with CT infection but did not do this for NG infection because the small number of NG infections was not sufficient to do a subgroup analysis. In our multivariate analysis, being an ethnicity other than Han was more likely to be at risk of CT infection as compared to Han ethnicity (AOR = 1.9). This ethnicity (mainly Dong, Miao and Yao) may be related to the rural-to-urban migration background which is related to a risk of acquiring STIs [24]. It has been documented that rural-to-urban migrants have an increasing risk to get STIs [24], but do have not sufficient reproductive health (RH) knowledge and utilization of RH services [25].

Regarding adverse pregnancy outcomes (APOs) of CT infection are concerned, our findings show that women who experienced any APOs (abortion, premature delivery, ectopic pregnancy or infertility) had 1.4 times higher risk of CT infection than those without any APOs. It has been generally recognized from previous serological studies that CT infection is strongly associated with ectopic pregnancy and other APOs [26–28]. Given the association of current CT infection with previous infection [29], our results further confirm the association between previous exposure to CT and APOs.

Association of inconsistent condom use with STIs has been documented in many studies [30, 31]. Given that our study focused on the general population, condoms were typically used for contraception purposes with regular partner. However, condom use is still critical in this population for preventing CT infection and other STIs because of a high proportion of men reporting to have multiple sex partners in China [11, 32].

In our study, another risk factor significantly associated with CT infection was self-report of reproductive tract symptoms (abnormal vaginal discharge and/or leucorrhoea increase in quantity) in the recent 3 months. Association of abnormal vaginal discharge with CT infections has been reported by many studies [33, 34], indicating that CT is one of the leading causes of clinical symptom of abnormal vaginal discharge. A study in which 6,150 patients with abnormal vaginal discharge were recruited from a hospital in Shanghai during 2011 and 2015 indicated5.0% abnormal vaginal discharges were caused by pathogen of CT [35].

The current study has several limitations. First, the study was conducted in one city with a special economic background in China and among women attending a local government-supported public welfare programme; any generalization of the results from this study should therefore be made with caution. Second, history in reproductive tract infection (RTI) symptoms was self-reported in our study; over or underreporting due to recall bias could impact the association estimates of this factor with infection. Overall, however, proportions of women who report abnormal vaginal discharge (6.4%) and leucorrhoea increase in quantity (6.6%) in our study were similar to many previous studies [36]. Third, detailed information on sexual behaviors were not systematically collected to investigate the associations of these factors with infection in our study. Reporting bias is a significant concern when collecting sexual behavior data as women are usually reluctant to divulge this information. In addition, reluctance to provide sexual behavioral information might deter participation in the study, compromising the participating rate.

Although these limitations should be addressed in the future studies, our findings could have significant implications for policy, practice and research. The high prevalence of chlamydial infection in our study population, particularly the women aged 20–29 years has called for urgent action for prevention and control of CT infection among young women in China. Given the fact that the majority of CT infections are asymptomatic [5] and a vaccine against CT infections is current unavailable [37], early and active detection of the infection followed by antibiotic treatment plays a crucial role in decreasing burden of the disease. Opportunistic screening for CT among young sexually active adults has been recommended in many high-income countries including the USA, the UK, Australia, Sweden, Denmark and Norway [38–40] but the UK is the only country to run the nationwide programme—the National Chlamydia Screening Programme (NCSP) [41], which targets all sexually active men and women under 25 years of age for annual chlamydia screening through various clinical and nonclinical settings. Modelling analysis based on literature review of asymptomatic sexually active women under 30 years of age has indicated the screening for CT to be cost effective at CT prevalence of 3.1%-10.0% [42]. Based on our findings, active screening for CT in our study population, particularly in women aged 20–24 years should be mostly cost-effective. However, further assessments of the acceptability, feasibility, efficacy, and cost-effectiveness of expanding screening for CT in general practice are required before a local or national recommendations could be made in China. In addition, an effortlessly collected, non-invasive and self-collected sampling approach and a commercially available, economically affordable and validated test, including point-of-care (POC) test, should be prerequisites to wider implementation of screening or testing [38, 39]. As part of these efforts, a pilot programme to expand active screening for CT to all sexually active women aged under 25 years in primary care settings (the Shenzhen Chlamydia Intervention Pilot, SCIP) was initiated in early 2017 in the country. First-hand findings from the pilot study will be useful for local and/or national government to design the evidence-based programme for reducing the burden of sexually transmitted infection in China.

In conclusion, CT infection is prevalent among women, particularly young women aged 20–24 years in Shenzhen, China. Thus, a comprehensive CT screening, surveillance and treatment programme particularly targeting this population would be justified and could reduce the burden of the infection in the country.

## Supporting information

S1 Data CT prevalence survey excel data. (XLSX)
