# Correction: Population-based study of chlamydial and gonococcal infections among women in Shenzhen, China: Implications for programme planning

**DOI:** 10.1371/journal.pone.0199907

**Published:** 2018-06-25

**Authors:** Zhen-Zhou Luo, Wu Li, Qiu-Hong Wu, Li Zhang, Li-Shan Tian, Lan-Lan Liu, Yi Ding, Jun Yuan, Zhong-Wei Chen, Li-Na Lan, Xiao-Bing Wu, Yu-Mao Cai, Fu-Chang Hong, Tie-Jian Feng, Min Zhang, Xiang-Sheng Chen

The affiliation for the 15th author is incorrect. Min Zhang is not affiliated with #2 but with #1: Shenzhen Nanshan Center for Chronic Disease Control, Shenzhen, China.
